# Similarity-Based Compression in Working Memory: Implications for Decay and Refreshing Models

**DOI:** 10.1007/s42113-023-00179-0

**Published:** 2023-08-03

**Authors:** Benjamin Kowialiewski, Benoît Lemaire, Sophie Portrat

**Affiliations:** 1 Department of Psychology, Cognitive Psychology Unit, University of Zürich, Binzmühlestrasse 14/22, 8050 Zurich, Switzerland; 2 Univ. Grenoble Alpes, CNRS, LPNC, 38000 Grenoble, France

**Keywords:** Working memory, Similarity, Compression, Computational modeling, TBRS

## Abstract

The ability to compress information is a fundamental cognitive function. It allows working memory (WM) to overcome its severely limited capacity. Recent evidence suggests that the similarity between items can be used to compress information, leading to a rich pattern of behavioral results. This work presents a series of simulations showing that this rich pattern of WM performance is captured using the principles of TBRS*, a decay and refreshing architecture. By assuming that similar items are compressed, the architecture can explain the beneficial effect of similarity on the items themselves. The architecture also explains the fact that when similar items are mixed with dissimilar items, this provides a proactive—but no retroactive—benefit on WM performance. In addition, the model captures fine-grained patterns of transposition errors recently reported. Several analyses are reported showing the robustness of the model’s predictions. We reached the conclusion that decay and refreshing theories provide a plausible explanation for compression effects in WM. These conclusions are discussed in light of recent experimental results. The importance of computational modeling for testing theories is emphasized.

## Introduction

The human mind is a powerful compression machine that can benefit from any underlying structure in what is perceived to compensate from its poor raw immediate memory. When regularities are present in the environment, humans can take advantage of them and summarize the core information in some way (Albrecht & Scholl, 2010; Alvarez, 2011; Ariely, 2001; Son et al., 2020). This ability to compress information has recently been shown to be important for the short-term retention of information. Studies in the working memory (WM) domain have shown that people can maintain only a limited amount of information (Cowan, 2001; Cowan et al., 2005). However, as soon as sequences contain compressible information, recall performance improves (Chekaf et al., 2016). Several studies have shown this direct benefit, in particular within the framework of chunking (Norris et al., 2020; Portrat et al., 2016; Thalmann et al., 2019). For instance, when presented with sequences such as “PDFCIARIP,” people can take advantage of their long-term memory knowledge to improve recall performance as compared to less compressible sequences, such as “XDRTFPLSC” (Chen & Cowan, 2005, 2009).

**Fig. 1:**
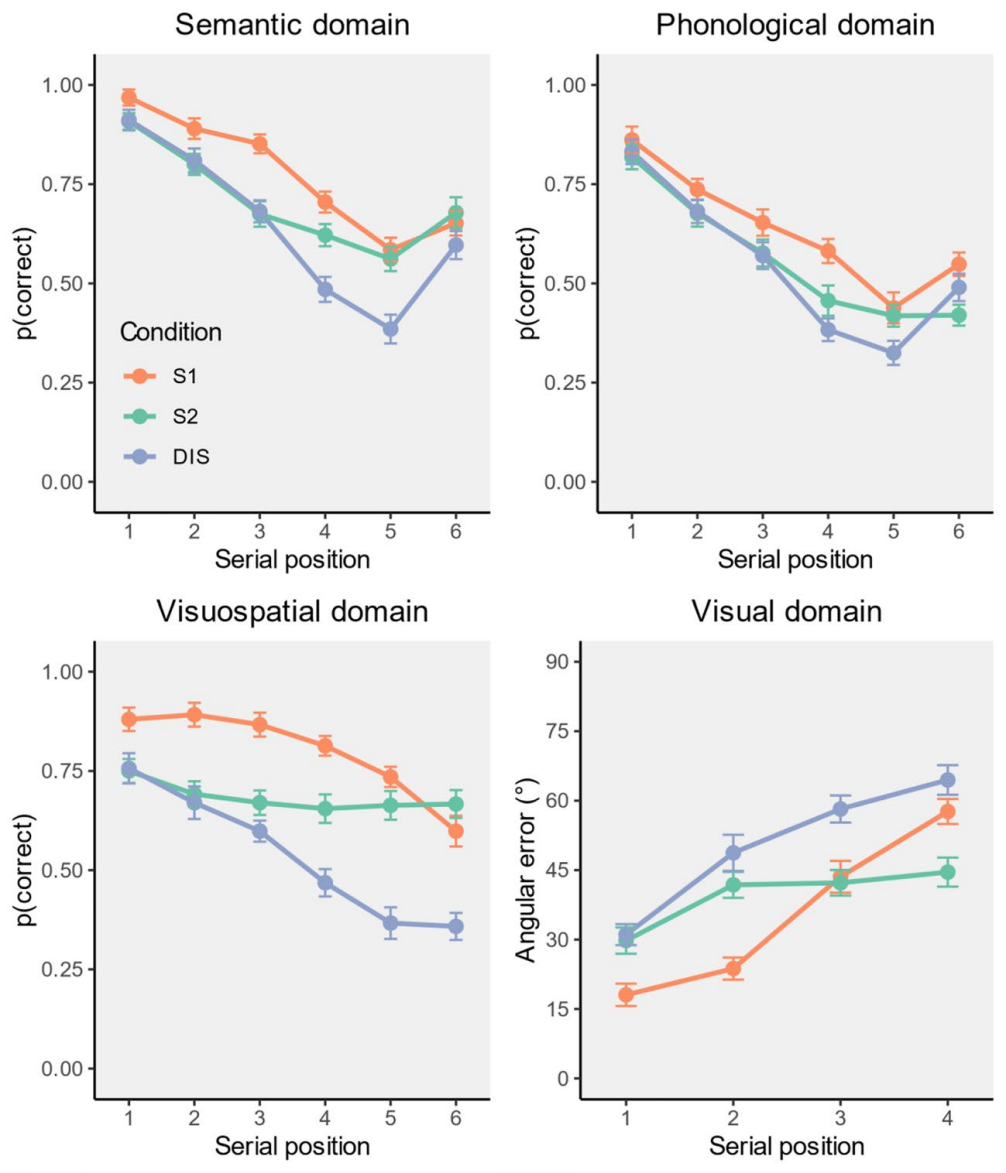
Proactive benefit of similarity observed across domains. *Note*. Memory performance in the visual domain was measured using a continuous color wheel. Performance in this task was therefore computed as the angular error from the target. S1 similarity in the first half of the list. S2 similarity in the second half of the list. DIS dissimilar

Recent evidence suggests that similarities between items can be used to compress information. When items are similar, they form a structure that is helpful for maintaining them in memory. For instance, in the list “piano, guitar, flute,” the semantic relationships between the items might be used to activate and use the superordinate category “musical instrument.” Similarly, in the list “cat, fat, mat,” people might activate the rhyme category “/æt/” and use it to maintain all items more easily. This recall advantage for similar vs. dissimilar items has been observed in the visual (Lin & Luck, 2009; Peterson & Berryhill, 2013; Quinlan & Cohen, 2012; Sanocki & Sulman, 2011), phonological (Fallon et al., 1999; Gupta et al., 2005; Nimmo & Roodenrys, 2004), visuospatial (De Lillo, 2004; Parmentier et al., 2005, 2006), and semantic (Monnier & Bonthoux, 2011; Neale & Tehan, 2007; Poirier & Saint-Aubin, 1995; Saint-Aubin & Poirier, 1999) domains, suggesting that this beneficial effect of similarity is a domain-general property of the human cognitive system. A recent work suggests an indirect benefit of compression (Kowialiewski et al., 2022b): when the first items of a list to be remembered are similar, they are obviously better recalled than dissimilar items. Most importantly, *subsequent* items, although dissimilar from each other, also benefit from being preceded by similar items. We will call this the *proactive benefit*. However, when the group of similar items are presented *after* the dissimilar items, the latter do not benefit from the similar group. In other words, there is an absence of *retroactive benefit*. This phenomenon is illustrated in Fig. 1. An example of the material for each domain used in Kowialiewski et al. (2022b) is reported in Appendix 1.

**Fig. 2:**
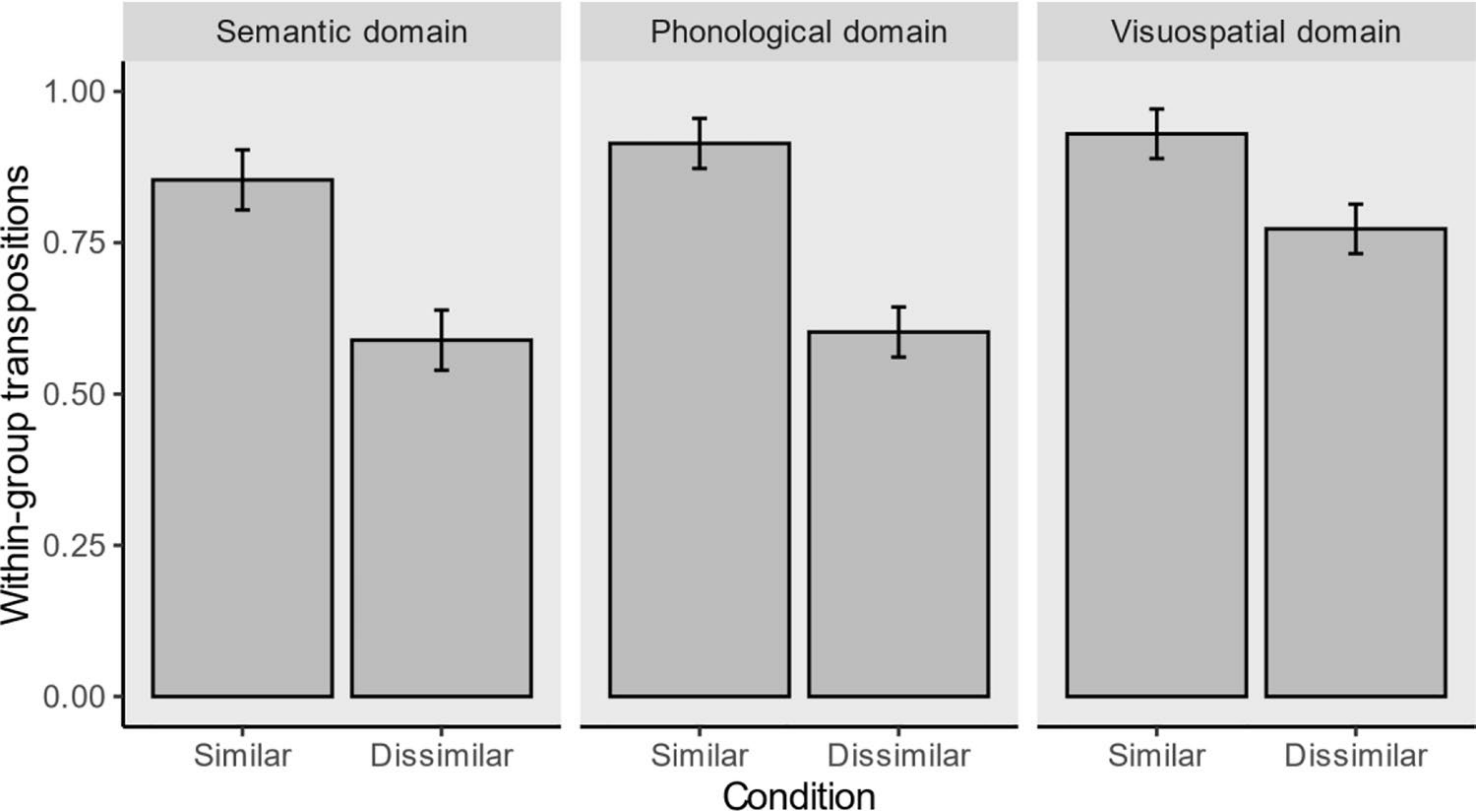
Within-group transpositions across the semantic, phonological, and visuospatial domains. *Note*. Within-group transpositions are computed by counting the number of times an item has been transposed toward another similar item position, divided by the total number of transposition errors in each condition. In the dissimilar condition, because transposition errors cannot be defined as being “within” or “between,” the proportions are computed as in similar conditions, by considering the same group of positions. Details of how this score was computed with examples are reported in Appendix 2

Another phenomenon related to compression has recently been observed when analyzing the position of recalled items (Kowialiewski et al., 2021a, 2022b). It has been shown that similarity constrains the pattern of *transposition errors*. Transposition errors refer to the displacement of an item to a wrong serial position. For instance, given the encoded sequence “ABCD” and the recalled sequence “AXBD,” item “B” has been transposed. When sequences are composed of similar and dissimilar items, similar items, when transposed, tend to stay within their group of similar items, rather than to move to groups of dissimilar items, and this when comparing the same positions in a completely dissimilar condition. In other words, when the to-be-remembered sequence contains similar items, there is an increase in *within-group transpositions* (see Appendix 2 for further details). To illustrate this, suppose the to-be-remembered sequence “S_1_S_2_S_3_D_1_D_2_D_3_,” where “S” and “D” refer to similar and dissimilar items, respectively. Results have shown that it is more likely to observe a transposition such as “S_1_S_3_S_2_D_1_D_2_D_3_” rather than “S_1_S_2_D_1_S_3_D_2_D_3_.” This phenomenon has been observed across the semantic, phonological, and visuospatial domains. The results from Kowialiewski and colleagues are illustrated in Fig. 2.^1^

One way to explain this set of results is by postulating the existence of a general compression mechanism that would provide the opportunity to better maintain other items. However, it is not trivial to understand how a compression mechanism may lead to proactive benefits, no retroactive benefits, and an increase in within-group transpositions. The aim of this study is to go beyond mere verbal descriptions of cognitive phenomena, and instead propose a computational model of the underlying cognitive processes explaining this domain-general property of the cognitive system.

One possibility regarding these patterns of results is that proactive benefits emerge from a reallocation of attentional resources following compression. According to the time-based resource sharing (TBRS) theory (Barrouillet et al., 2004), items encoded in WM constantly decay when out of attention. However, the deleterious impact of decay can be counteracted using the focus of attention, a central bottleneck limited to one process at a time. The attentional focus allows the decaying representations to be refreshed, provided there is enough free time to do so. In TBRS, WM capacity is therefore constrained by the constant balance between refreshing and decay. Importantly, the focus of attention is supposed to be a domain-general attentional mechanism acting on any domain. When framed through the TBRS model, the beneficial effect of compression is straightforward. Since WM load is reduced following compression, this frees up some time that can be devoted to refreshing more items. Accordingly, this free time should benefit the non-compressed items, which should be better recalled. When coupled with a compression mechanism, this model has the potential to explain the fact that similar items tend to be transposed within their group: if three items have been compressed, they are more likely to be recalled together, which mechanically reduces the probability to confuse them with other, dissimilar items.

Although these predictions from the TBRS model are intuitive, their plausibility remains to be formally established. In this regard, computational models are fruitful tools that can help researchers to think about theories (Farrell & Lewandowsky, 2010). Such a formal implementation of the TBRS model exists, and is called TBRS* (Oberauer & Lewandowsky, 2011). This architecture was initially implemented to account for human performance in complex span tasks. It has been shown to be able to reproduce several benchmark phenomena observed in WM, including cognitive load effects, serial position curves, and error types (i.e., transposition errors, omissions, extra-list intrusions). Subsequent extensions of this model successfully captured other effects, such as interference (Lemaire & Portrat, 2018). Adaptations of this architecture have successfully modeled chunking effects in verbal WM (TBRS*C, where the letter “C” stems for “chunk,” Portrat et al., 2016) and semantic similarity effects (TBRS*S, where the letter “S” stems for “semantic,” Kowialiewski et al., 2021b).

### The Present Study

Inspired by these computational models, our approach aims at challenging decay/refreshing models regarding their capacity to reproduce behavioral similarity effects that are shared by all domains. We consider this as a critical challenge, because previous studies suggested decay and refreshing models as being unable to account for compression effects in WM. For instance, in a recent study, Thalmann et al. (2019) manipulated the presence of chunks at different serial positions. Their study showed similar observations as ours: chunking proactively but did not retroactively impact memory performance. They claimed that decay and refreshing models mispredict the absence of retroactive impact of compression (i.e., when the chunk is presented toward the end of the list):

*Although the benefit of chunks is also assumed to be larger when presented first, a decay-and-rehearsal theory nevertheless predicts that chunks presented last should help memory for earlier presented lists. This is because a single chunk representation requires less time to be rehearsed than the representations of three individual consonants. Hence, even though a chunk should help more for not-chunked information when presented first, it is also assumed to help when presented last.* (Thalmann et al., (2019, p. 11).

However, without a formal test of the theory using computational modeling, this interpretation of decay and refreshing models remains vague and imprecise. The present study aims at solving this issue. In the next section, we briefly describe the TBRS* architecture, followed by our new model which keeps the core principles of TBRS*. We then assess the plausibility of this architecture to account for the general pattern of results associated with similarity effects described in the present study. Another goal of the present study is to perform a detailed analysis of the model’s parameters to understand its behavior in a fine-grained manner. This detailed diagnosis allows us to understand the fundamental properties of the TBRS* architecture, and thus to make gains at the theoretical level.

It is important to note that we do not seek to explicitly describe the compression mechanisms operating in WM. These mechanisms have recently been the object of an extensive description elsewhere (Norris & Kalm, 2021). The details of these mechanisms would require modeling how information is represented in each domain, which is beyond the scope of the present study. Instead, the purpose of the following simulations is to assess the plausibility of decay models to account for the similarity effects we presented in this introduction in a general manner. Finally, in no way this work advocates in favor of the theoretical superiority of the TBRS* architecture over other models. Other explanations are also plausible and have been discussed elsewhere (Kowialiewski et al., 2022b).

## Method

### TBRS*: General Description

**Fig. 3:**
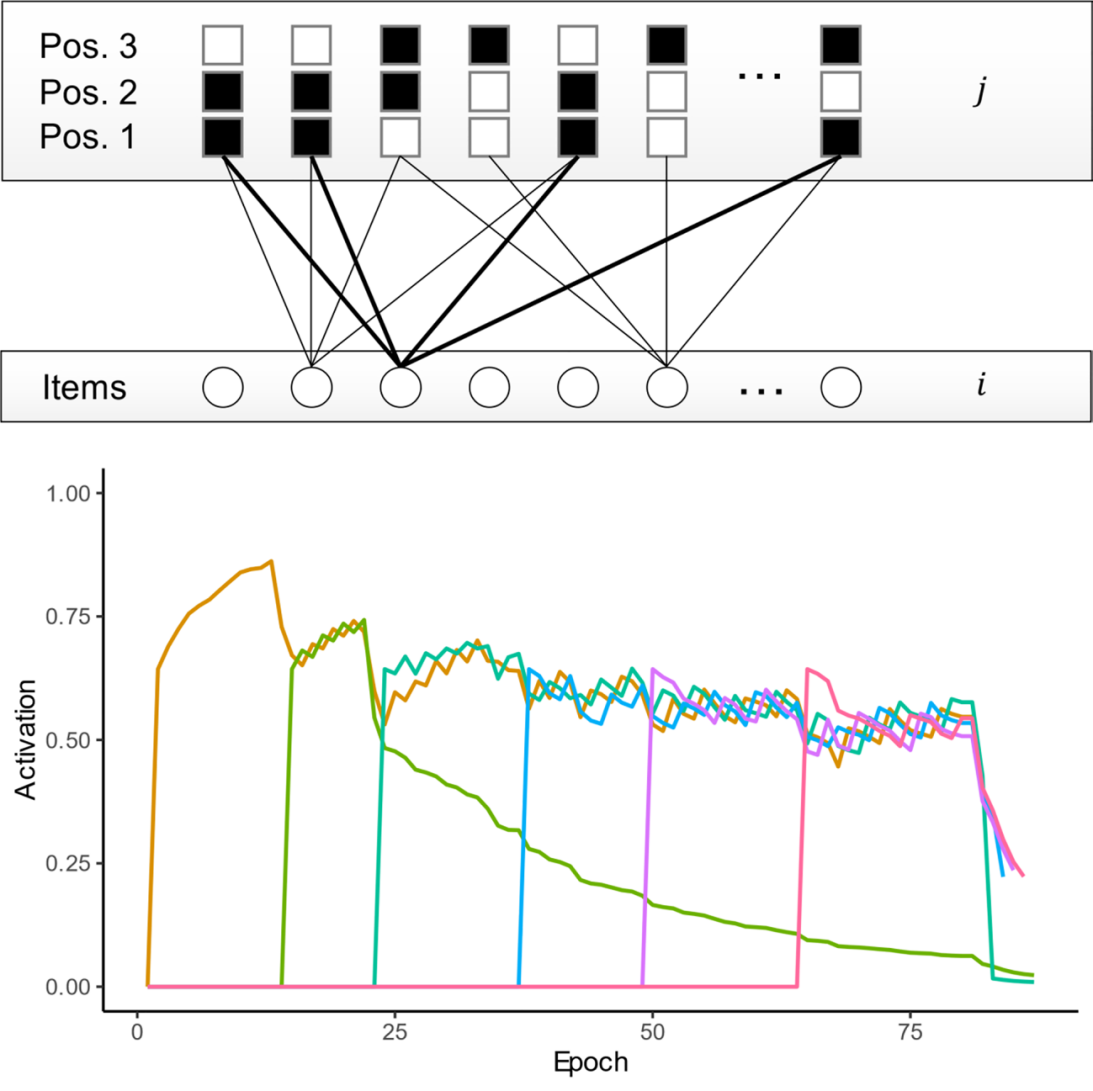
General overview of the TBRS* architecture. *Note*. Upper panel: illustration of the TBRS* architecture. Items are kept in memory via item-position associations. It is those associations that constitute the WM content. The item-position associations constantly decay when out of attention. To counteract the deleterious effect of decay, those associations are restored through refreshing. Lower panel: activation values of the model over one trial. The burst that follows each encoding attempt illustrates the maintenance period of the model, during which items are rapidly refreshed using the focus of attention. Items can be lost during maintenance. This happens in this trial, as shown by the green item which progressively decays

The TBRS* architecture (Oberauer & Lewandowsky, 2011) is a fully interconnected two-layer neural network (see Fig. 2, upper panel). The first layer is an item layer, in which items are represented by localist units. The second layer is a positional layer, in which the serial positions are represented in a distributed fashion. Adjacent positions share a proportion of positional nodes, and this proportion decreases exponentially as between-position distance increases. Encoding in TBRS* is performed by creating item-position associations through Hebbian learning. These item-position associations constantly decay. To counteract decay, the item-position associations are maintained via refreshing, using the focus of attention. Refreshing in TBRS* is performed by first retrieving the original item by activating the relevant position nodes and selecting the most activated item. The item is then re-encoded through Hebbian learning. Importantly, in TBRS*, refreshing operates on only one item at a time, as assumed by a one-item focus of attention (Barrouillet et al., 2004; Nee & Jonides, 2013; Oberauer, 2009). After the encoding and maintenance phases, the items are recalled. Recall is performed by retrieving the items during the refreshing period, by cueing the positional layer. Once recalled, the item is removed from memory by suppressing its item-position associations, a mechanism also called response suppression (Lewandowsky, 1999). The dynamics of TBRS* are illustrated in Fig. 3, lower panel.

Errors in TBRS* occur from three main sources: (1) transposition errors due to the overlap between positional markers, (2) omission errors when an item activation falls below the retrieval threshold, and (3) intrusion errors. The main source of errors comes from a Gaussian noise that is added to all item activations at retrieval. The presence of omission errors is a particularly critical feature of the TBRS* architecture. As items need to be constantly maintained, omission errors may eventually occur during the inter-item maintenance intervals, before recalling the items. This is particularly likely to occur as the maintenance demand increases; if too many items need to be maintained, the balance between decay and refreshing breaks up, and an item is eventually dropped from the competition. This is illustrated in Fig. 3, where item 2 is no longer maintained by the model and progressively decays.

### Modeling Inter-item Similarity Benefit

TBRS* needs to be supplemented with a mechanism to reproduce the way people take advantage of inter-item similarities. We assumed that when an item is encoded, a high similarity with the previous item tends to associate them. This association can be extended to more than two items to form a group. However, this grouping is not systematic and occurs with probability PCR (probability of chunk retrieval), to account for the fact that participants can miss the grouping opportunity. If grouping still takes place, compression is then achieved by considering that the members of a group of associated items are processed simultaneously rather than separately. They are all refreshed and recalled at the same time. In the verbal TBRS theory, refreshing is a sequential process that reactivates items one at a time, although it is unclear how the scheduling is performed (Vergauwe et al., 2016). Early proposals assumed a sequential refreshing schedule as in verbal rehearsal (Tan & Ward, 2008) but a more recent one suggested that refreshing would focus on the least activated items first (Lemaire et al., 2018). Still, a group of associated items refreshed in one shot, instead of separately, would mechanically increase the likelihood that the other items would be refreshed. Indeed, in the competition for refreshing, items are likely to be refreshed more often if the number of candidates is lower. This directly explains the inter-item similarity proactive effect discussed previously. This compression mechanism can also be used to explain the benefit of chunking letters into pre-existing acronyms (Portrat et al., 2016). It can also be interpreted in terms of resource reallocation: refreshing episodes can be viewed as temporal resources that are shared among items. Consequently, refreshing a group of associated items at once, instead of individually, amounts to a resource reallocation process over the other items. We now present the computational model in more detail.

### TBRS*: Mathematical Description

Items and contexts in the model are represented in a localist and distributed fashion, respectively. Units in the item and context layers can take two values: 0 or 1. When an item is presented, its localist unit in the item layer becomes activated (i.e., setting its value to 1). Similarly, nodes in the contextual layer also become active (i.e., setting their values to 1). The core WM representation in the model is stored in a weight matrix ${w}_{i,j}$, which contains all newly formed associations between an item $i$ and its context $j$.We now detail how these representations are formed.

#### Encoding

In the original TBRS* model, when a new memorandum is presented, it is encoded by increasing each connection weight ${w}_{i,j}$ between the corresponding item $i$ in the item layer and the nodes $j$ in the position layer. For example, if the first memorandum is F, all links between the item node F and the different nodes coding for position 1 increase. This increase follows an exponential law over time bounded by an asymptote $L$, chosen such that the maximal possible activation of a memorandum is 1. The slope of the exponential increase is controlled by the variable *r*. The increase in weight ${w}_{i,j}$ during a duration $t$ is thus given by:

1 $${\Delta w}_{ij}=(L- {w}_{ij})(1-{e}^{-rt})$$

$r$ is obtained by sampling a random draw from a normal distribution centered around $R$, with a standard deviation of 1. This random draw makes the encoding process noisy. The original value of $R$ is defined by considering that memoranda such as letters are supposed to be well encoded after 500 ms (Jolicœur & Dell’Acqua, 1998). Reaching 95% of maximal activation in 500 ms gives $R=6$. In our simulations, $R$ is however considered as a free parameter, because some items (such as words) are likely to be more weakly encoded than others (such as letters).

#### Compression

The novel aspect of our implementation is the addition of a probability to detect the similarity between the current item and the preceding ones, determined by a parameter that we call PCR (probability of chunk retrieval). Basically, after encountering a series of similar items, the model has a probability PCR to detect the similarity between items. If this occurs, the items are grouped together for the duration of the trial. Once grouped, the associated items are refreshed and recalled together, which means that their connections in the weight matrix ${w}_{i,j}$ are reinforced in parallel.

#### Decay

When attention is devoted to another item, all connection weights decay over time ($t$) following an exponential law modulated by a decay rate $D$:

2 $${w}_{ij(t+\Delta t)}= {w}_{ij(t)}.{e}^{-D\Delta t}$$

#### Refreshing

As long as there is time before the next memorandum appears, refreshing takes place to counterbalance the deleterious effect of decay. To be refreshed, an item has to be retrieved from memory first. For this, the same equations as described for recall are used (see below). Instead of refreshing the items in a cumulative fashion as in the original model, our version selects the least activated item and refreshes it (see Lemaire et al., 2018 but also Kowialiewski et al., 2021b). This refreshing schedule has been shown to produce the most realistic fit to behavioral data and is a key factor to explain the proactive benefit in the context of decay and refreshing models. If an item is part of the group of associated items, all members are refreshed in parallel. Refreshing an item is performed by strengthening each of its links to its current position nodes following the same formula as encoding (Eq. 1), but for a shorter period defined by parameter ${T}_{r}$. The refreshing rate $R$ is the same as for encoding. Next, the least activated item is searched and refreshed, and so on until a new event stops the refreshing steps.

#### Recall

At the end of the task, recall is carried out by retrieving the items one by one in their original order. This is done by cueing the item with its position, and then selecting the most associated item to that position j, which is defined as:

3 $$selectedItem={argmax}_{i}\left({\sum }_{j=1}^{n}{w}_{i,j}+n\right)\;where\;n \sim N(0,\sigma )$$

The zero-centered Gaussian noise *n*, drawn from standard deviation $\sigma$, is added to simulate retrieval errors. However, no item is retrieved if the activation level of the selected item is below the threshold parameter $\theta$. During recall, the WM representations continue to decay normally following Eq. 2 to account for output interference effects (i.e., memory performance degrades as more items are recalled, see Cowan et al., 2002 for a demonstration of this phenomenon).

#### Joint Recall Mechanism

In addition to the PCR parameter as described above, we considered an additional mechanism to the original TBRS* architecture. When retrieving an item which is part of the group of associated items, the current and subsequent positions are filled in with the members of the group with probability PJR (probability of joint recall). The rationale behind this mechanism is that recalling an item which is part of a grouped structure should necessarily help to retrieve other items from the same structure. In other words, when an item part of a chunk is retrieved, other members of this chunk are also retrieved, because they are part of the same representation. Without it, the model would behave as if items were merely retrieved individually, without being supported by the chunk. However, this global retrieval does not contain the order in which to assign elements to positions. Therefore, all permutations are considered and the one that maximizes the association with the positions is selected. Each permutation of items ${i}_{0}\dots {i}_{k}$ is assigned a score which is the sum of the activation value of each element with respect to its position:

4 $$score\left({i}_{0}\dots {i}_{k}\right)= \sum\nolimits_{k}(\sum\nolimits_{i}{w}_{ik,j+k}+n)$$

where *j* is the current recall position and $n \sim N(0,\sigma )$. For instance, if the group pear-apple-plum was identified at encoding and plum is recalled in position 3, the permutation plum-pear-apple is assigned the sum of the weights between plum and position 3, plus the sum of the weights between pear and position 4, plus the sum of the weights between apple and position 5. The scores of all other permutations (pear-plum-apple, pear-apple-plum…) are computed and the one with the highest score is considered. If no group was identified at encoding or if the random draw with probability PJR fails, recall is performed as usual. Each individual recall takes a duration defined by the parameter ${T}_{rec}$ to account for the duration of a response.

### Parameter Estimation

The purpose of the simulations was to test whether a decay and refreshing architecture can account for the qualitative pattern of results presented in the introduction. The goal was therefore to reproduce effects and not specific behavioral data. Our parameter estimation procedure can be decomposed into two steps.

First, the original TBRS* architecture has several basic parameters controlling its behavior, listed in Table 1. These parameters, by themselves, cannot model compression effects. Among those basic parameters, four of them were varied freely (i.e., $R, \theta ,D, \mathrm{and}\;P)$. We chose to specifically estimate these parameters because they are the most likely to vary from one experimental setup to another. For instance, the processing rate $R$ most likely varies from one material (i.e., letter) to another (i.e., words in our experiments). It is therefore important to re-estimate its value. Those parameters were identified using a simulated annealing algorithm (French & Kus, 2008; Kirkpatrick et al., 1983) to minimize the root mean squared error (RMSE) between the model’s outcome and the experimental data. This estimation was based on the serial position curves of Experiment 1 from Kowialiewski et al., (2022a, b),^2^ in the condition in which all the items were semantically unrelated. This implies that the compression parameters (i.e., PCR and PJR) were deactivated at this stage (i.e., setting their values to zero). The list of fixed and free parameters and associated values are available in Table 1.

**Table 1:** Fixed and free parameters of the models. The free parameters were estimated using a simulated annealing algorithm

Fixed parameters				
Parameter	Meaning	Value		
$\sigma$	Noise added at retrieval	0.01		
$s$	Standard deviation of processing rates	1		
$Te$	Mean duration of an encoding episode	0.5		
$Tr$	Mean duration of a refreshing episode	0.08		
$Trec$	Mean duration of a recall episode	0.5		
$n$	Number of items in long-term memory	81		
$ISI$	Inter-stimulus interval	1.5		
Free parameters				
Parameter	Meaning	Minval	Maxval	Best
$R$	Processing rate	1.0	9.0	**2.062**
$\theta$	Retrieval threshold	0.1	0.5	**0.143**
*D*	Decay rate	0.05	0.9	**0.271**
*P*	Overlap between positions	0.1	0.9	**0.414**

Bold values represent the best-fit parameters

**Fig. 4:**
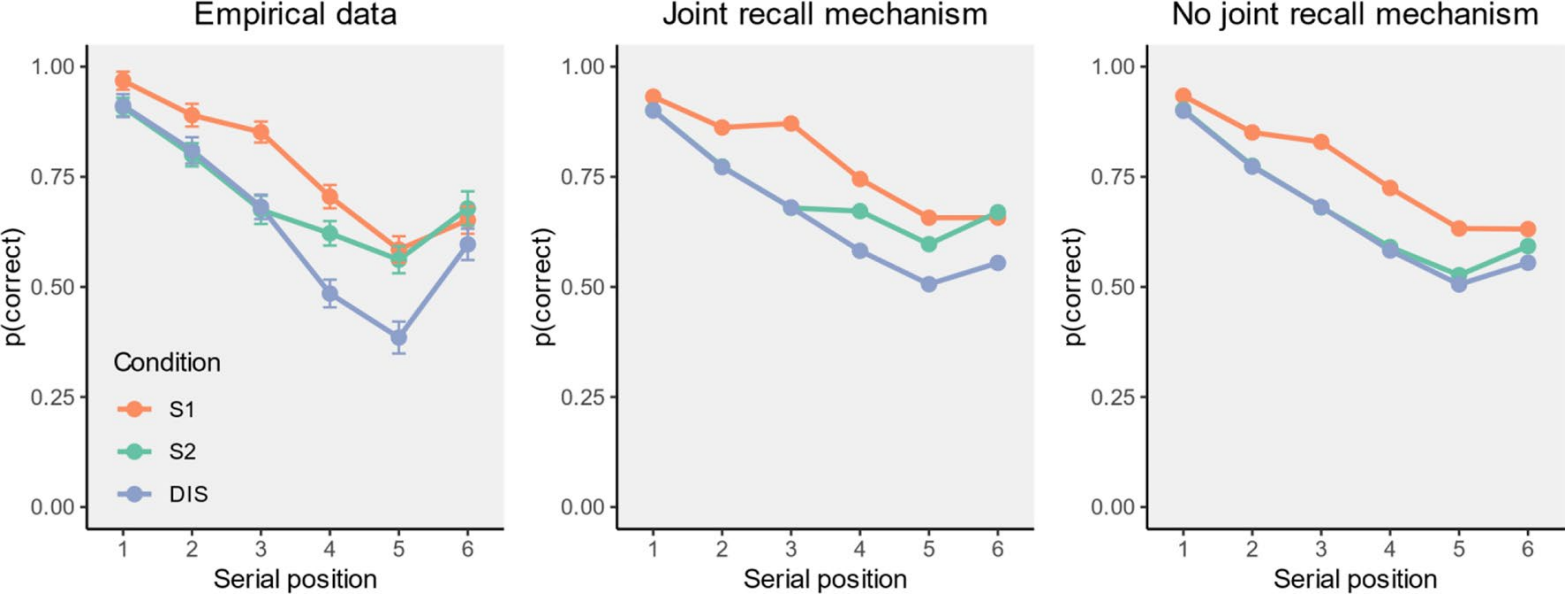
Model’s outcome with and without the joint recall mechanism. *Note*. Recall performance across serial position for each similarity condition. Left panel: empirical data. Middle panel: simulations with the joint recall mechanism. Right panel: simulations without the joint recall mechanism. S1 similarity in the first half of the list. S2 similarity in the second half of the list. DIS dissimilar

Second, after we identified the model’s original parameters, we fixed these parameters across all experimental conditions. This latter procedure helps to avoid overfitting. To simulate compression effects, we estimated the parameter values of the two compression mechanisms (i.e., PCR and PJR) using a grid search, specifically using the two experimental conditions involving similar items. As we will see in the result section, the expected effects are robustly observed with a large set of parameter values. In fact, all those parameter values significantly contributed to our phenomena of interest, and this in a reliable and predictable manner. The graphs illustrating the model’s behavior were plotted with a value of 0.9 for both compression parameters (see Fig. 4).

### Simulation Results

This result section is divided into several parts. We first show the main simulation results regarding similarity effects and transposition errors. We then explore the compression parameters and their impact on the model’s behavior. We also performed an overall diagnosis of the model to understand why it behaves the way it does. We then test the generality of the model’s behavior, by exploring a large range of parameters and how these parameters influence WM performance.

#### Overall Performance

Results averaged over 10^5^ simulations are displayed in Fig. 4, left panel. First, the model (middle and right panels) produces a reasonably good quantitative fit to the empirical data (left panel) in the dissimilar condition (RMSE = 0.047). Second, the model successfully reproduces the classical serial position curves, with primacy and recency effects. Third, the model also captures the impact of compression: compressed items are better recalled compared to non-compressed items. Fourth, and most importantly, the model successfully captures the proactive impact of compression, with items following similar triplets being associated with higher WM performance. Finally, there was also an absence of retroactive impact.

An interesting aspect of the model is related to the joint recall mechanism we implemented. As can be seen in Fig. 4, right panel, when this mechanism is deactivated (i.e., setting PJR to 0.0), the triplet in the second half of the list (S2) has only a negligible impact on recall performance. This is particularly the case in position 4 (i.e., the first item of the triplet). This result is explained by two main factors. First, triplets in the S2 condition have an overall lower probability (*p* = 0.636) to be recognized as a chunk during maintenance compared to triplets in S1 (*p* = 0.858). This is because compression first requires the WM representations that compose the triplet to be robust enough. In the S2 condition, these representations are less likely to be available: their activation level is weaker because they benefit less from refreshing compared to items in S1. Second, triplets in S2 are also less likely to be available at recall because these items suffer the most from output interference; i.e., they are recalled last and suffer the most from decay. At recall, the triplet is therefore more likely to be forgotten, even though it has been encoded in its compressed form. The additional joint recall mechanism plays a critical role, by restoring the original WM representations.

#### Within-Group Transposition Errors

**Fig. 5:**
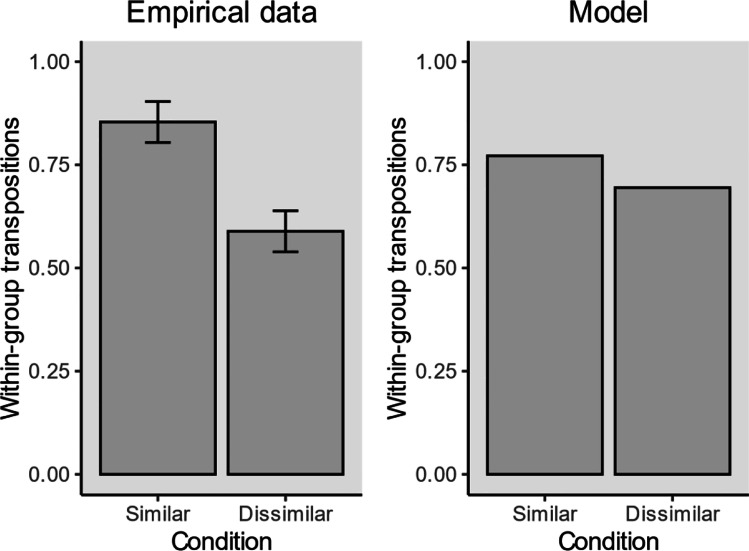
Within-group transpositions. *Note*. Left panel: empirical data. Right panel: simulations. See Appendix 2 for the details of how this score was computed, with examples

The model captures the increased within-group transposition errors in similar conditions, compared to the dissimilar condition. This effect, illustrated in Fig. 5, is the direct product of the compression parameter: compressed items tend to be recalled together, rather than with other non-compressed items of the list. For instance, when recalling the sequence “SSSDDD,” if the three “S” have been recognized as a whole, they will be recalled together. This in turn reduces the probability that one of these items will be transposed outside of the group. Note that transposing items outside of the group can still happen. Indeed, the results in Fig. 5 show that the effect is relatively modest compared to what happens in humans (see Fig. 2). These results nonetheless support the idea that the increased transposition errors induced by similarity may partially depend on the joint activation of similar items (Kowialiewski et al., 2021a, 2022b; Parmentier & Maybery, 2008). In the next section, we performed a more thoughtful exploration of the role of each compression parameter.

#### Exploring the Compression Parameters

**Fig. 6:**
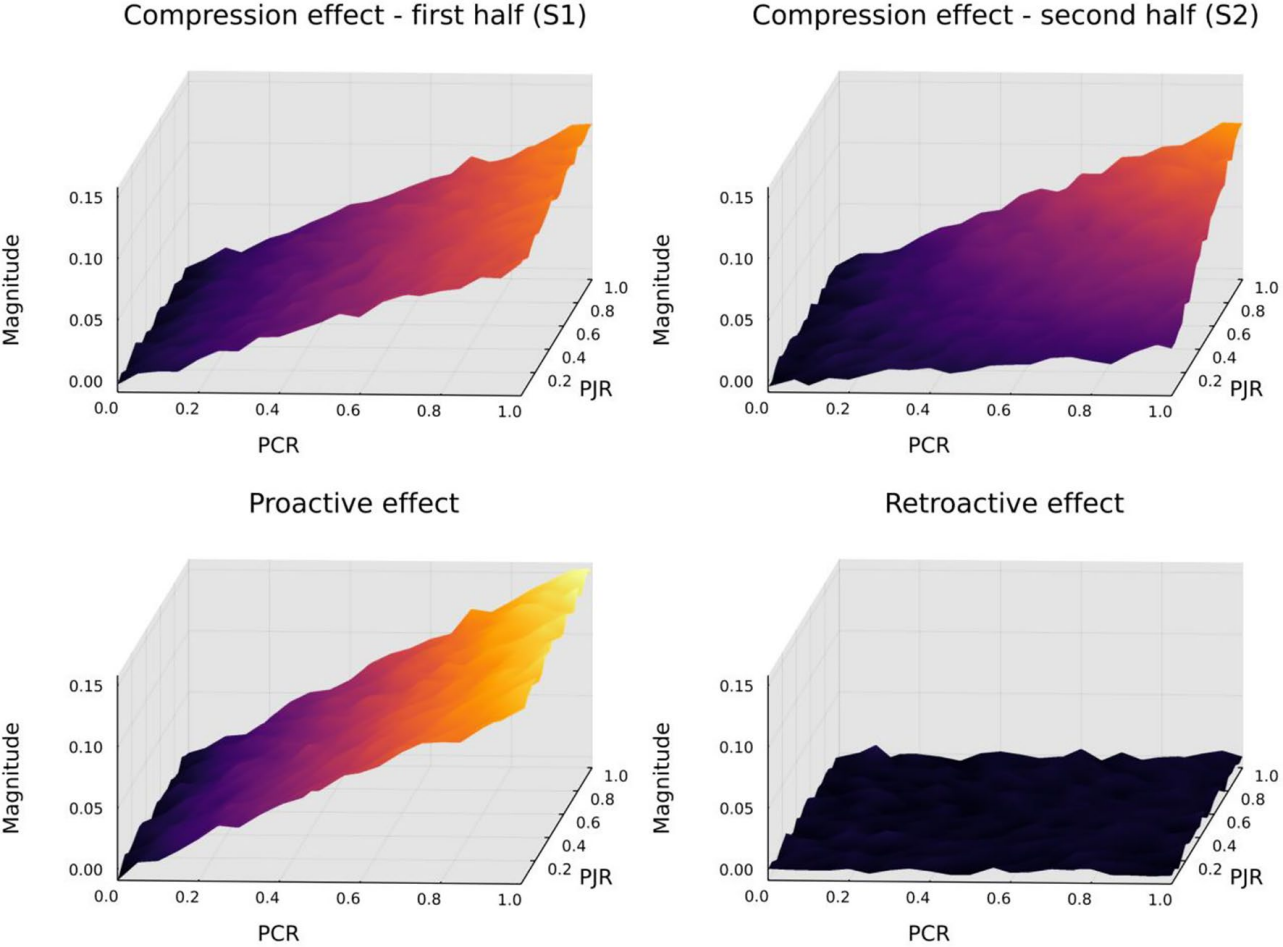
Exploration of the impact of the compression parameters. *Note*. The magnitude of each effect was computed by averaging the difference between the condition of interest and the non-compressed condition. For instance, the upper left panel was computed by subtracting recall performance between the S1 and DIS conditions, averaged over positions 1 through 3. The brightness of the surface represents strength of magnitude specific to each effect of interest. Slight error variations are caused by the non-deterministic property of the model. PCR probability of chunk retrieval, PJR probability of joint recall

Figure 4 suggests that the two compression parameters, PCR and PJR, may contribute differently to the effects we observed in the empirical data. Specifically, PJR appears to contribute mostly to the recall advantage for the triplet itself in S2, but not in S1. Similarly, when the joint recall mechanism is deactivated, there is still an obvious proactive impact of the triplet in S1, compared to NS. This suggests that the locus of the proactive impact is mostly driven by PCR that operates when the memoranda have to be processed. This intuition is confirmed by Fig. 6, in which we investigated the impact of the two compression parameters on recall performance using a grid search considering a range of 21 values for each parameter, which is a total of 441 parameter sets explored. Each parameter set was estimated through 25,000 simulations. The surface represents the magnitude of each effect of interest (similarity effects in the first and the second half of the lists, proactive and retroactive effects) as a function of the two parameters. The brighter locations over the surface represent a stronger impact of compression on recall performance. First, the recall advantage for triplets in S1 compared to DIS is mostly driven by the PCR parameter (see upper left panel). Second, the two parameters appear to significantly contribute to the specific impact of compression in S2 (see upper right panel). When PCR is set to zero, the impact of compression is non-existent. This is intuitive: if no item has been compressed, no beneficial effect can be observed at all. Third, the proactive effect is also driven exclusively by the PCR parameter (see bottom left panel). This is because the proactive benefit of compression builds up during maintenance thanks to the reallocation of refreshing opportunities. Since PJR impacts WM performance locally at recall, this parameter cannot produce a proactive effect. Finally, it can be seen that whatever the compression parameters, the model always predicts that compression has no obvious retroactive impact on WM performance, as indicated by the black surface in the bottom right panel. This suggests that the lack of retroactive impact of compression in our model is robust.

#### Proactive Effect

**Fig. 7:**
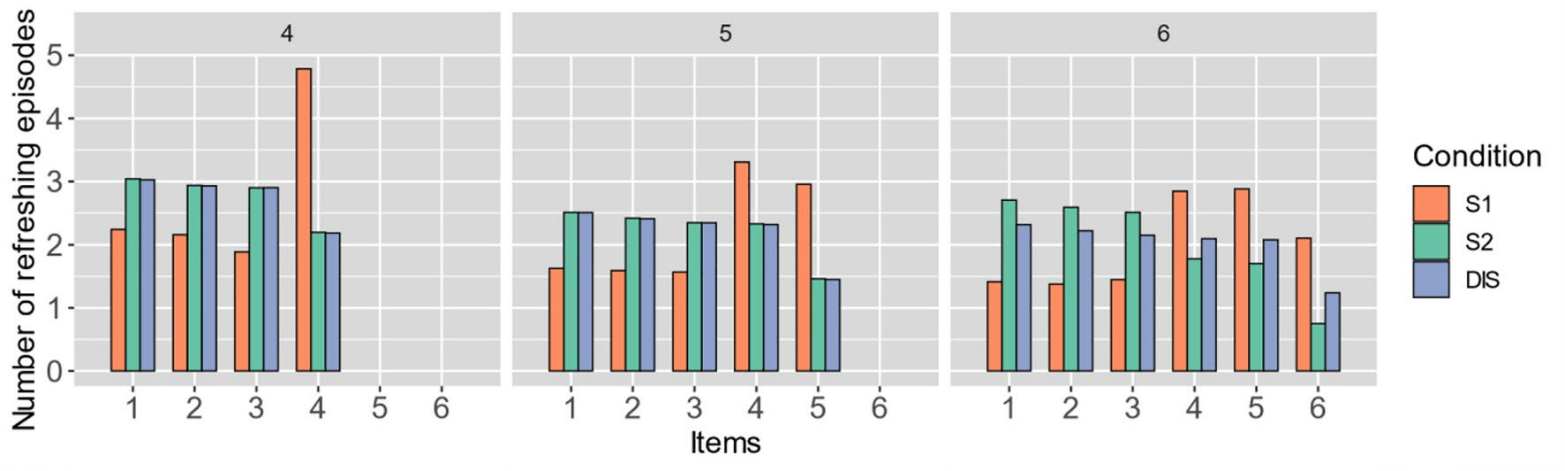
Distribution of the refreshing episodes over positions. *Note*. Mean number of refreshing episodes (across 100,000 simulations) over the different items for each semantic condition (S1, S2, DIS). Each panel (numbered 4, 5, and 6) represents the maintenance phase that directly follows the encoding of a given item. For instance, panel 5 represents the maintenance phase between the encoding of item 5 and item 6

One critical aspect of the simulation is the observation of a proactive benefit. This proactive benefit is due to a reallocation of refreshing opportunities. Figure 7 displays the number of refreshing attempts over a whole trial, averaged over 10^5^ simulations. Each panel represents the inter-item retention interval that follows each encoding period. It is during this retention interval that the items can be maintained through refreshing. For simplicity, we only displayed the last three retention intervals, as they are the most informative. As can be seen, in the S1 condition, when compared to the DIS condition, the number of refreshing episodes is reallocated toward items 4, 5, and 6. This happens because the items that compose the triplets in positions 1–3 are refreshed in a single shot. Hence, fewer refreshing attempts are required to keep them active. Instead, these refreshing attempts are redirected toward the other, non-compressed items. This in turn creates a proactive benefit on recall performance, by preventing the subsequent items to be lost during the retention intervals.

#### An Absence of Retroactive Impact

**Fig. 8:**
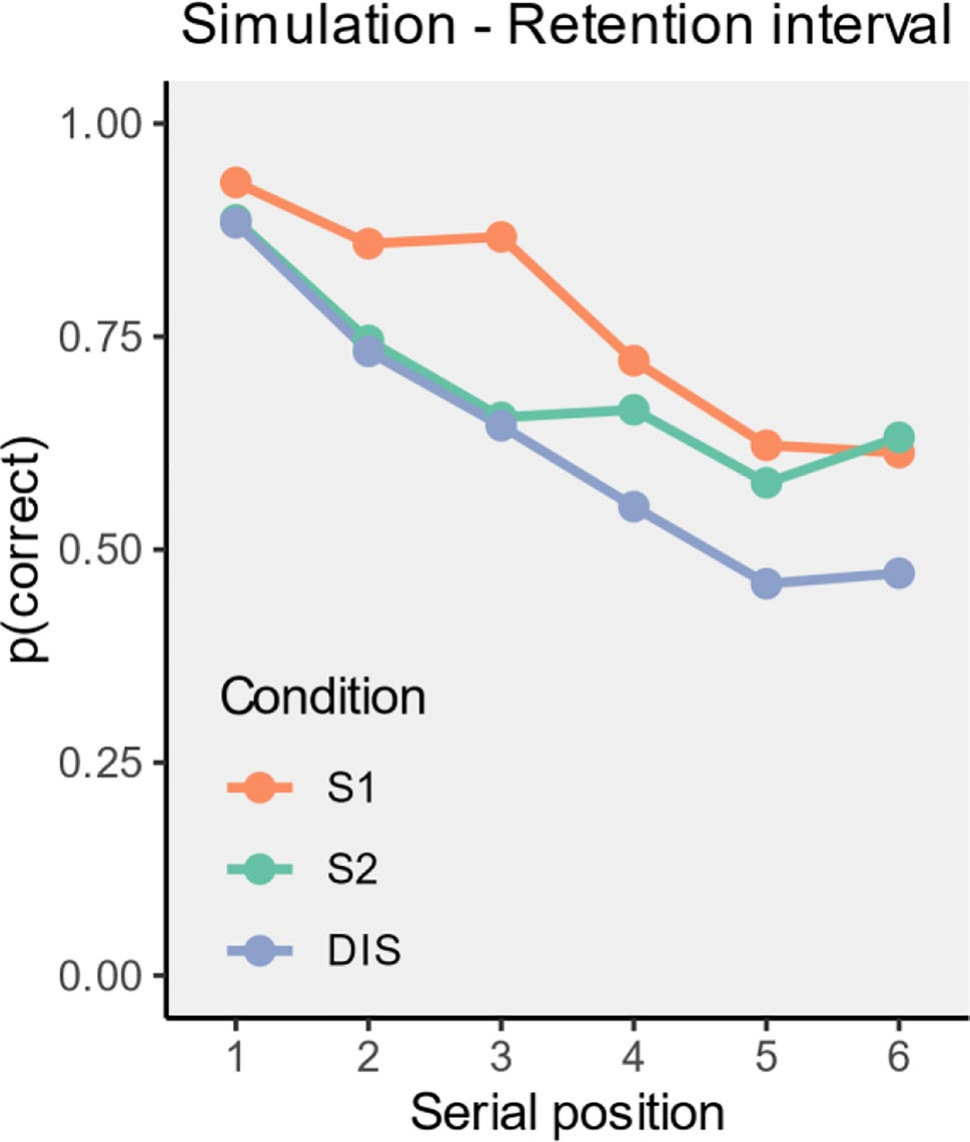
Model’s behavior after addition of a 5-s retention interval. *Note*. The parameters were the same as those used in the original simulations (see Fig. 4)

**Fig. 9:**
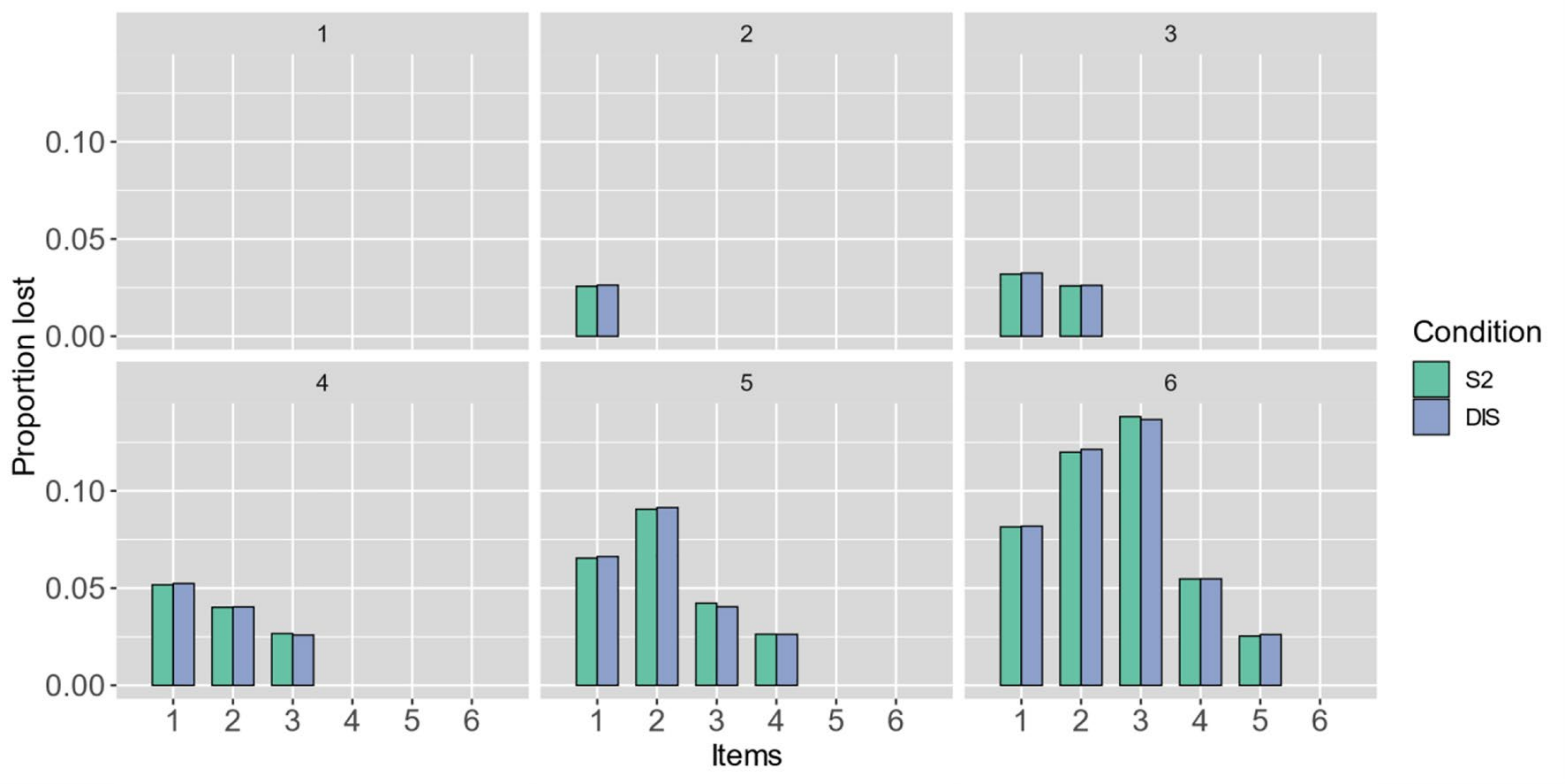
Proportion of trials for which a given item has been lost. *Note*. Results are shown for the S2 and DIS semantic conditions. The results are averaged across 100,000 simulations. Each panel represents the maintenance phase that directly follows the encoding of an item

In Fig. 7, it can be seen that the number of refreshing episodes in S2 is also somewhat reallocated toward items 1, 2, and 3 when compared to DIS. How does it come that these items do not benefit from this reallocation process, as it occurs in the S1 condition? It could be argued that this is because the compression mechanism occurs much later (i.e., at the end) in S2, which provides little room for the attentional reallocation to build up. We explored this possibility by adding a retention interval of 5 s before each recall phase. The addition of this retention interval did not produce a substantial retroactive impact, as reported in Fig. 8. One critical thing must be understood as regards the TBRS* architecture. With the current set of parameters, the model can only maintain a limited number of items at the same time, i.e., around 3–4 items. When this limitation is reached, one or more items have to be dropped from the competition due to WM overload. This phenomenon is illustrated in Fig. 9, where the number of trials in which the items have been lost (i.e., activation level below the retrieval threshold) averaged over 100,000 simulations. As we move on to the list, more and more items are lost during the retention intervals. As shown, when the triplet is presented at the end of the list (S2), no item is saved. This is because the reallocation of refreshing opportunities cannot save the items that are already lost. If an item has already been forgotten, it cannot be refreshed, because retrieval is a necessary condition for refreshing. This is clearly illustrated in Fig. 9, where the proportion of items lost in panel 6 does not decrease after the triplet has been encoded in S2 compared to DIS.

**Fig. 10:**
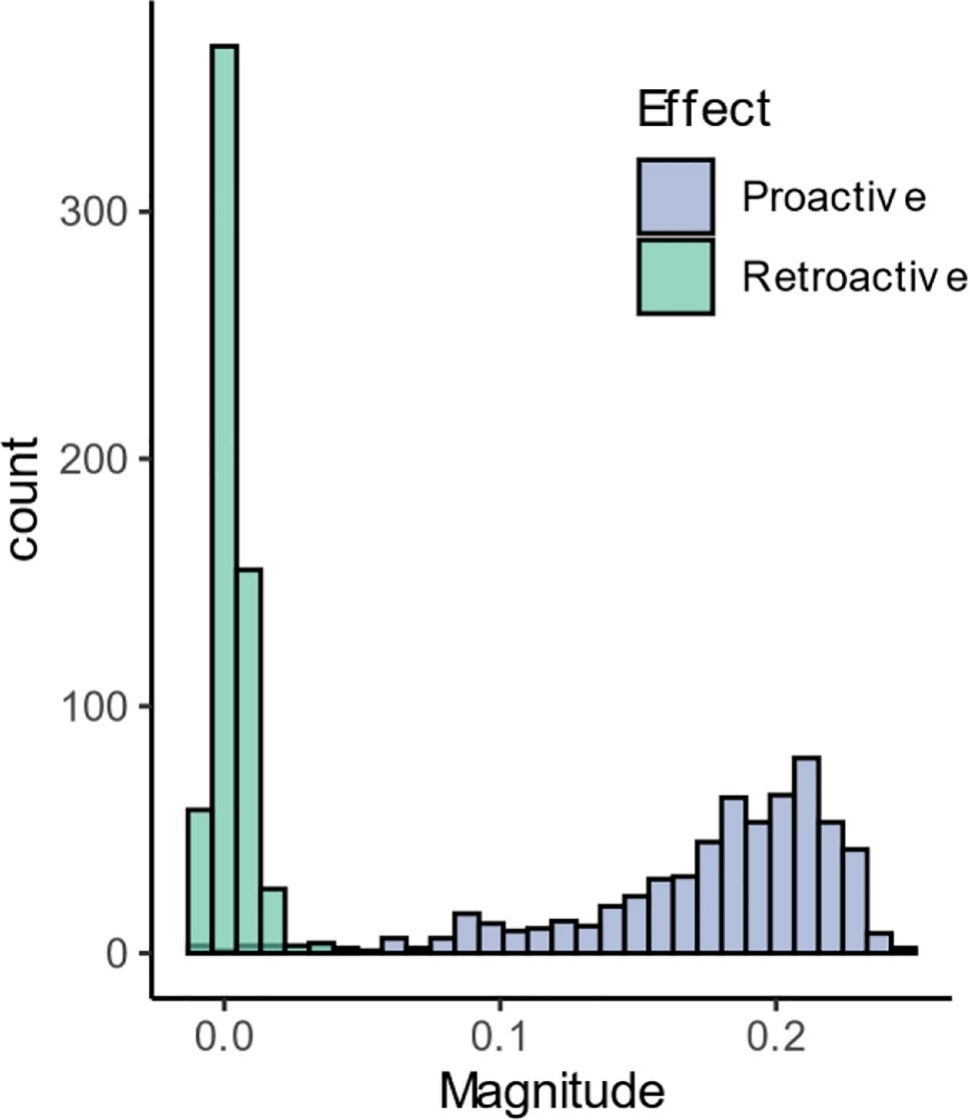
Distribution of the proactive and retroactive effects. *Note.* Distribution of the magnitude of the proactive (in blue) and retroactive (in green) impact of compression across a wide range of parameter values. The magnitude is computed here using the averaged difference between the DIS condition and the S1 (positions 4 through 6) and S2 (positions 1 through 3) conditions for the proactive and retroactive effects, respectively

The simulation results we presented so far show an obvious absence of the retroactive effect of a compressible sequence of memoranda. However, this result may not be the general rule of decay and refreshing models. Instead, it could be argued that this absence of retroactive impact is specific to the set of parameters we found when fitting the model using the simulated annealing algorithm. To test this possibility, we ran a grid search across a range of plausible values for the TBRS* basic parameters. We then assessed to what extent these parameters produced a retroactive impact when activating the compression parameters specific to our model. The technical details of this analysis are reported in Appendix 3. Figure 10 represents the distribution of the magnitude of the difference associated with each data point included in the grid search. As can be seen in green, the data points associated with the retroactive impact are massively centered around zero (median = 0.002, *P*_95%_ = [− 0.006; 0.014]). This means that the model hardly produced a retroactive impact. In contrast, the model produced a proactive effect in a consistent manner (median = 0.187, *P*_95%_ = [0.074; 0.225]), as shown by the blue bars. These results demonstrate that the predictions drawn from our simulations presented above are robust and are not merely the by-product of the specific parameters we chose for the model.

## Discussion

In this study, we assessed the plausibility of decay and refreshing models to account for the pattern of results observed when between-item similarity is manipulated in immediate serial recall tasks. Basic results show better recall performance for similar vs. dissimilar items. Importantly, the similar items proactively—but not retroactively—enhance recall performance for other, dissimilar items. The group structure furthermore constrains the pattern of transposition errors, by increasing the probability that an item migrates toward the position of another similar item, rather than a dissimilar item in the same list. Our model, assuming similar items as being compressed, was able to predict these patterns of results to a varying degree.

### How Does Compression Impact Working Memory Performance?

The important results of the present study were the ones showing that a decay and refreshing architecture can capture the proactive benefit of similarity, and the absence of retroactive benefit. These results are explained by the compression mechanism we assumed to occur in the context of immediate serial recall: when people detect the presence of redundancies in the flow of information, they can group similar information and process it as one unit, which helps the maintenance process. This assumption is based on a series of work showing consistently higher WM performance for similar vs. dissimilar material (Chekaf et al., 2016; Mathy & Feldman, 2012). In these studies, a compression metric based on the degree of redundancy in the information can be defined, and reliably predicts WM performance. This beneficial effect of similarity is a well-replicated phenomenon, observed across a wide variety of experimental conditions (Morey, 2018; Ramzaoui & Mathy, 2021).

Coupled with the assumption that WM capacity is limited by decay and refreshing, the proactive benefit observed following compression is straightforward and intuitive: since all similar items are refreshed in a single shot when grouped together, this leaves more opportunities to refresh the other, dissimilar items. The lack of retroactive effect observed in TBRS* simulations is however less intuitive. We find it better illustrated with a more concrete example, as we already did in a previous publication (Kowialiewski et al., 2021b). Suppose that your task is to juggle a bunch of balls, but your expertise allows you to juggle three balls at most. In this example, the balls represent the items, gravity represents decay, and throwing a ball in the air is the equivalent of refreshing. Each time an item is encoded, someone is throwing a ball at you. At the beginning, it is relatively easy to juggle up to three balls. However, each time a new ball is added to the competition, the load becomes too high, and the system is unstable. Consequently, one ball must be dropped to stabilize the system again. In this case, the number of balls one can juggle does not change and is equivalent to about three. Now suppose that you can throw three balls in the air at once, because these balls become agglutinated thanks to a magnetic force. This is what happens when items are compressed, which leaves a lot of time to juggle with the remaining balls that one will throw at you. This creates a proactive benefit in the model. Now suppose the balls become agglutinated only after the 5th or 6th have been thrown. Chances are that some balls have already been dropped from the competition due to the high load imposed by the task. In this case, having the balls agglutinated at the end will not help to save those balls that have already been dropped. This illustrates the reason why the presence of triplets or chunks does not retroactively impact WM performance in TBRS*. Retrieval is therefore a necessary condition for refreshing, which differs from what is happening in other models implementing refreshing. In TBRS*, if items are no longer available in WM during the between-item intervals, they cannot be retrieved and therefore they are not refreshed. In contrast, models such as the Primacy Model (Page & Norris, 1998) or the Revised Feature Model (Saint-Aubin et al., 2021) assume a refreshing mechanism as restoring WM representations, regardless of the extent to which the information is degraded.

An interesting aspect of our simulation is related to the recall advantage for compressed information when the triplet appeared in the second half of the list. Without the addition of a joint recall mechanism which helps to recall the compressed information from partial information, this advantage was anecdotal. This is explained by the fact that similar items in the second half of the list are less likely to be compressed, but also because they are less often recalled due to output interference. The addition of the joint recall mechanism critically overcame this disadvantage.

Our decay model is not the only one able to explain the pattern of results described in this study. Norris et al. (2020) recently showed that the absence of retroactive benefit when lists contain chunks can be captured using the simple mechanistic principles of the Primacy Model. In this model, serial order is coded through a linearly decreasing gradient of activation. To recall items serially, the model starts by selecting the most activated position which is the first one in most cases (but not always due to a noisy retrieval process). That position’s activation is then set to a lower value (i.e., response suppression), and the model moves on to the next most activated marker, which is more likely to be the second one, and so on. In their adaptation of the Primacy Model, chunking was implemented by assuming that items composing the chunk are treated as singletons. Their model predicted a recall advantage for chunked vs. non-chunked items, because chunked items are recalled together: if at least one item from the chunk is recalled (or omitted), all the other items in the chunk are also recalled (or omitted). This mechanism is related to the joint recall mechanism implemented in the present study. Interestingly, they also discussed the absence of retroactive impact at a theoretical level. The original Primacy Model assumes items as being reactivated through cumulative rehearsal. An important feature of this model is that rehearsal stops when all items in the list cannot be properly rehearsed during the between-item interval, which is based on empirical observations (Tan & Ward, 2008). Hence, having compressed items at the end of the list does not necessarily predict the retroactive benefit, because rehearsal already stopped at this point. This shows again that decay and refreshing models offer a plausible explanation for compression, contrary to previous claims (Thalmann et al., 2019). Importantly, this plausibility depends on the assumptions underlying the model. It is therefore incorrect to rule out a whole family of models based on a particular implementation.

The grouping mechanism we implemented in the TBRS* architecture successfully captured the increased within-group transpositions as observed in the empirical data. This effect appears because compressed items, when recalled, are more likely to be recalled together rather than separately. Compression is therefore a plausible mechanism. This compression mechanism works regardless of the cognitive architecture used. Similar patterns of results have been observed by Parmentier and Maybery (2008) using the ACT-R architecture, in which several grouping manipulations (temporal, spatial, and pitch-based) were modeled using a hierarchical representation. They showed that this grouping mechanism successfully predicted overall recall performance in grouping manipulations, as well as patterns of transposition errors and recall latencies.

### The Problem of Representing Information in Working Memory

One specific aspect of our modeling approach is that the items’ features are not explicitly represented. Due to this lack, similarity per se is not implemented, nor how similarity can lead to compression. Mechanisms through which similarity can lead to compression have been proposed in the literature. One example comes from works assuming colors as being represented across a pattern of neural population (Nassar et al., 2018; Wei et al., 2012). In this approach, neurons coding for similar representations reinforce each other through local recurrent excitation, and global inhibition occurs between all neurons to keep the neuron’s activity in a stable state. Neural activity can sometimes merge due to the local recurrent excitation, and this is likely to be the case as the to-be-remembered items are more similar to each other. This in turn reduces the number of representations that the model needs to maintain. Although plausible, this mechanism is however specific to the visual domain.

In contrast to these previous studies, we are agnostic regarding which compression mechanisms take place in WM. Instead, we merely assume compression as occurring, which is implemented probabilistically in the PCR parameter. Actually, the way information is represented in WM most probably varies substantially from one domain to the other, which can be detected in the variability of similarity effects across domains. For instance, semantic similarity does not seem to induce similarity-based confusion on memory for order (Poirier & Saint-Aubin, 1995; Saint-Aubin & Poirier, 1999 but see Ishiguro & Saito, 2020 for a different interpretation). At the same time, similarity-based confusions in the semantic domain are reliably observed in single probe recognition tasks (Atkins & Reuter-Lorenz, 2008), but only when lists are semantically similar (Cowan et al., 2022). This contrasts with other domains, such as the phonological one, for which similarity-based confusion is systematically observed independently of the task at hand (Baddeley, 1966; Jalbert et al., 2008; Lin & Luck, 2009; Visscher et al., 2007). In addition, the effect of phonological similarity has recently been shown to be more complex than we previously thought (Roodenrys et al., 2022). Since the effects of similarity vary from one domain to another, it is likely that the way information is represented across different domains differs as well. Including a way to represent information would have the consequence of enhancing the model’s predictive power. It would also allow the simulation of continuous responses, which is widely used in the visual domain (Bays, 2009; Zhang & Luck, 2008). At the same time, this would also have the consequence of adding free parameters to the model and increasing its complexity. Not adding further assumptions in the model comes with a gain in terms of generalizability. For the sake of parsimony, we therefore decided to be agnostic regarding the way information is represented in WM. The strength of our simulations is to show that simple rules can globally account for relatively complex phenomena across domains.

### Decay Models in Perspective

The fact that memory is subject to time-based forgetting is an intuitive one and corresponds to a long tradition in the memory literature (Peterson & Peterson, 1959; Ricker et al., 2014). In the field of WM, there is still a strong debate about the existence or not of this temporal decay. In the past years, there was a ping-pong game between publications that attempted to prove the existence of temporal decay (Barrouillet & Camos, 2009; Barrouillet et al., 2011; Portrat et al., 2008), and studies showing its absence and advocating the unique role of interferences to explain forgetting (Lewandowsky & Oberauer, 2015; Lewandowsky et al., 2009; Oberauer & Lewandowsky, 2013, 2014).Our work does not pretend to show the theoretical superiority of decay-based models over other families of models. Other alternative explanations are also plausible and have been discussed extensively in some of our previous works (Kowialiewski et al., 2021a, b, 2022a, b). What our results show is that a given phenomenon initially thought to be unable to be predicted by decay-based models (Thalmann et al., 2019) can be captured. This however requires to be tested beyond a naïve interpretation of the theory and with the help of computational modeling. When a model becomes complex, which happens as soon as a few variables interact over time, its implementation and simulation become unavoidable. Beyond the problematic aspects of decay-based models, we think that the series of simulations we presented here can be useful for general theories of WM. The present work only explores the boundary conditions of decay and refreshing models, hoping that it would shed further light for researchers when interpreting their results.

### Future Directions and Challenges

This study serves as a proof of concept, showing that a decay and refreshing architecture can explain compression effects in WM. However, several questions remain unanswered. Among those, the exact mechanisms through which similarity can lead to a compressed representation. Some authors suggest that this could be achieved by extracting a summary representation of the items (Alvarez, 2011; Ariely, 2001). It is still however not clear how such a mechanism would prevent a loss of information for individual items. Moreover, recent studies have shown that presenting similar items in an interleaved manner (i.e., alternating similar and dissimilar items) diminishes the beneficial effect of similarity (Kowialiewski et al., 2021a, 2022a; Saint-Aubin et al., 2014). These findings pose a challenge for compression-based models as they introduce complexity to the dynamics of potential compression mechanisms. Overall, this study presents a promising approach to explaining compression effects in WM using a decay and refreshing architecture. However, there are still unanswered questions regarding the underlying machinery of compression mechanisms.

## Conclusion

Through a computational approach, the present study showed that a WM model based on a time-based decay phenomenon associated with a refreshing mechanism can capture robust effects related to the similarity between information. Although some issues remain as to how information is represented and compressed in WM across different domains, it seems clear that computational modeling becomes a necessary tool to test cognitive theories, especially when their complexity increases.

## Appendix 1

In these experiments, stimuli were always presented sequentially. This appendix provides examples of different sequences in each domain, for each experiment condition.

### Semantic Manipulation

**S1** bus, camion, voiture, soeur, ail, neige [bus, truck, car, sister, garlic, snow]

**S2** coussin, vautour, prêtre, bouche, palet, salive [pillow, vulture, priest, mouth, palate, saliva]

**DIS** lac, lèvre, hyène, saturne, facteur, couette [lake, lip, hyena, saturn, postman, duvet].

### Rhyming Manipulation

**S1** malette, galette, palette, boulot, prime, poire [briefcase, cake, pallet, work, bonus, pear]

**S2** mars, veste, siège, balade, salade, malade [mars, jacket, seat, walk, salad, patient]

**DIS** voile, malette, raison, cuite, peste, source [sail, briefcase, reason, hangover, plague, source]

## Appendix 2. Details of the within-group transposition score

The proportion of within-group transpositions was computed for each condition by dividing the number of transposition errors corresponding to a displacement within the same group of similar items by the total number of transposition errors. When similar items are presented in the first half of the list (i.e., S1 condition), a within-group transposition corresponds to a transposition occurring between items 1, 2, and 3. When similar items are presented in the second half of the list (i.e., S2 condition), a within-group transposition corresponds to a transposition occurring between items 4, 5, or 6. The condition in which no similar item was presented (i.e., DIS condition) served as a control. In this condition, within-group transposition errors were computed by using the same grouping pattern as the condition of reference. For instance, if participants recalled “ACBDEF” and “ABDCFE” in the S1 and DIS conditions, the within-group transpositions associated with these two sequences would be 2/2 (i.e., two transpositions in positions 1 through 3, two occurring within the same group) and 0/1 (i.e., one transposition occurring in positions 1 through 3, zero occurring within the same group), respectively. Proportionalizing for the number of transposition errors controls for the fact that people are expected to produce more transposition errors in some conditions than others (e.g., when items are phonologically similar vs. dissimilar).

## Appendix 3

To assess the robustness of the absence of retroactive impact in the model, we first performed a grid search over the TBRS* basic parameters, and this across a range of plausible values. Those values are reported in Table 2. In this grid search, 8 values were considered for each of the 4 different parameters, leading to an exploration of 4096 sets of parameter values in the model. A total of 10,000 simulations were run for each set of parameter values. Among those 4096 sets of parameter values, we considered only those that produced realistic patterns of WM performance across serial positions. These patterns include the production of a primacy effect (i.e., decreased recall performance across positions 1 through 5) over the item and strict serial recall criteria, and the omission rate. We did not consider the recency effect, as some of our experiments such as the visuospatial task did not produce a recency effect. We furthermore discarded the sets of parameters that produced ceiling and floor effects, which were defined as recall performance superior to 0.8 and inferior to 0.4, respectively. This whole selection process led to the inclusion of 614 sets of parameter values, for which we then assessed the impact of compression across the S1 and S2 conditions. This impact of compression was assessed using a value of 0.9 for both PCR and PJR.

**Table 2:** Range of parameters explored in the grid search

Parameter	Meaning	Lower bound	Upper bound	Step
*R*	Processing rate	1.0	4.0	0.429
θ	Retrieval threshold	0.1	0.25	0.021
*D*	Decay rate	0.0	0.6	0.086
*P*	Overlap between positions	0.1	0.6	0.071

## Footnotes

1 Note that due to the continuous report of colors, transpositions could not be computed in a straightforward manner in the visual experiment reported by Kowialiewski and colleagues.

2 The choice to fit the model against the data from the first experiment is arbitrary. It does not matter which set of data we fit the model to. This is because all experiments conducted by Kowialiewski and colleagues produced the same pattern of results, as already shown in Fig. 1.

## Declarations

### Open Science Statement

All the data and codes have been made available on the Open Science Framework: https://osf.io/yut4v/. This study was not preregistered.
